# A Deep Learning Algorithm for Multi-Source Data Fusion to Predict Effluent Quality of Wastewater Treatment Plant

**DOI:** 10.3390/toxics13050349

**Published:** 2025-04-27

**Authors:** Shitao Zhang, Jiafei Cao, Yang Gao, Fangfang Sun, Yong Yang

**Affiliations:** School of Automation, Hangzhou Dianzi University, Hangzhou 310018, China; 221060387@hdu.edu.cn (S.Z.); 221060395@hdu.edu.cn (J.C.); 221060388@hdu.edu.cn (Y.G.); 221060389@hdu.edu.cn (Y.Y.)

**Keywords:** industrial effluent treatment plant, data-driven, deep learning, effluent prediction, multi-source data fusion, sustainability, machine learning

## Abstract

The operational complexity of wastewater treatment systems mainly stems from the diversity of influent characteristics and the nonlinear nature of the treatment process. Together, these factors make the control of effluent quality in wastewater treatment plants (WWTPs) difficult to manage effectively. To address this challenge, constructing accurate effluent quality models for WWTPs can not only mitigate these complexities, but also provide critical decision support for operational management. In this research, we introduce a deep learning method that fuses multi-source data. This method utilises various indicators to comprehensively analyse and predict the quality of effluent water: water quantity data, process data, energy consumption data, and water quality data. To assess the efficacy of this method, a case study was carried out at an industrial effluent treatment plant (IETP) in Anhui Province, China. Deep learning algorithms including long short-term memory (LSTM) and gated recurrent unit (GRU) were found to have a favourable prediction performance by comparing with traditional machine learning algorithms (random forest, RF) and multi-layer perceptron (MLP). The results show that the R^2^ of LSTM and GRU is 1.36%~31.82% higher than that of MLP and 9.10%~47.75% higher than that of traditional machine learning algorithms. Finally, the RReliefF approach was used to identify the key parameters affecting the water quality behaviour of IETP effluent, and it was found that, by optimising the multi-source feature structure, not only the monitoring and management strategies can be optimised, but also the modelling efficiency of the model can be further improved.

## 1. Introduction

Nowadays, with the rapid development of industrialisation and urbanisation, resulting in increasingly serious pollution of water resources, especially wastewater issues have become a major environmental challenge facing the world. Pollutants in wastewater, such as organic matter, nitrogen, phosphorus, and other harmful substances, can lead to environmental issues like water quality deterioration and eutrophication if not properly treated before being discharged into water bodies [1]. These problems can negatively impact aquatic ecosystems and human health. For instance, organic pollutants consume large amounts of oxygen during decomposition, resulting in oxygen depletion and aquatic organism mortality [2,3]. Moreover, excessive nitrogen and phosphorus can trigger algal blooms, causing hypoxia and affecting aquatic species survival [4]. Some pollutants may even accumulate in the food chain, posing a threat to human health [5]. Wastewater treatment is not only related to environmental protection, which directly affects human health and quality of life, but also affects the sustainable development of cities. Therefore, it is extremely important to improve the efficiency of wastewater treatment and to ensure the stability and sustainability of the process. Therefore, enhancing the efficiency of wastewater treatment and ensuring the stability and sustainability of the treatment process are of paramount importance. These measures play a crucial role in reducing the pollutants discharged into the aquatic environment. To achieve these goals, a basic and necessary condition is the modelling and prediction of key effluent quality indicators [6,7,8,9].

In modern WWTPs, maintenance work requires the continuous monitoring of key water quality indicators such as pH, conductivity, chemical oxygen demand (COD), ammonia (NH_3_-N), total nitrogen (TN), and total phosphorus (TP). Among them, indicators like pH and conductivity are detectable through online real-time monitoring systems. Yet, the gathering and analysis of crucial metrics such as COD, NH_3_-N, TN, and TP prove demanding due to their higher time and cost requirements. This time requirement associated with sampling and testing is a major disadvantage in ensuring water quality standards are met and efficiently managed in WWTPs [10]. Concurrently, these indicators are closely correlated with water pollution. COD reflects the organic pollutant content in water by measuring the oxygen consumed during chemical oxidation of organic matter. A higher COD value indicates more severe organic pollution [11]. NH_3_-N, TN, and TP are associated with eutrophication pollution [12]. Excessive levels of these nutrients can cause water eutrophication, triggering algal blooms. This reduces water transparency, affects submerged plants’ photosynthesis, and upon algal death and decomposition, consumes dissolved oxygen, potentially causing aquatic organism mortality due to oxygen depletion [4]. This disrupts aquatic ecological balance and alters biological community structures. The accurate prediction of these effluent water quality indicators can optimise the wastewater treatment process, provide early warnings of potential pollution risks, and support decision making for effective pollutant control in water environments. This is of vital importance for reducing the water pollution and protecting aquatic ecosystems.

Recently, many researchers have worked on developing effective models for predicting the quality of effluent water from WWTPs. These models fall into two types: mechanistic water quality prediction models and data-driven water quality prediction models. Mechanistic water quality models are based on microbial kinetic equations to predict water quality [13,14,15], and although they have some guiding significance in design and operation, they are often limited in practical application due to high complexity, large data requirements, weak generalisation, high computational costs, and strict requirements for professional knowledge [16,17,18]. With technological advances, wastewater treatment facilities have become more advanced, enabling the online monitoring of multiple source variables and the accumulation of a large amount of data, which promotes data-driven techniques in modelling applications and will overall improve the efficiency of data utilisation.

Data-driven water quality prediction models can effectively address real-time measurements of key indicators in wastewater treatment. As indirect measurement tools, these models can predict difficult-to-measure variables online and adapt to real-time control needs. For example, some models rely on linear assumptions, while others employ more sophisticated nonlinear or machine learning methods to capture complex patterns and relationships in the data [19]. For example, some linear regression models can effectively handle large amounts of wastewater water quality data that may have multiple covariates, while preventing overfitting and improving the generalisation of the model [20]. This is crucial for the real-time pre-treatment of various water quality parameters in WWTPs, especially in environments with complex and highly variable datasets.

Recently, machine learning has gained significant attention for its ability to effectively process nonlinear and complex data. This technology has been widely applied in predicting effluent quality in wastewater treatment plants. Machine learning models can capture complex nonlinear relationships between system variables and address multicollinearity issues by analysing large amounts of historical and online data, without requiring a deep understanding of the underlying physical mechanisms. Numerous studies have demonstrated the effectiveness of various machine learning methods in water quality prediction. Lu et al. proposed two hybrid decision tree-based machine learning models for short-term water quality prediction that showed good stability [21]. Abouzari Milad et al. systematically evaluated 12 linear and nonlinear regression models and conducted an in-depth comparison of their performance in predicting chemical oxygen demand (COD) in wastewater treatment plant effluents, highlighting the efficiency and robustness of machine learning approaches in handling complex wastewater data [22]. Additionally, Yang Chong et al. proposed an adaptive dynamic nonlinear partial least squares (PLS) model incorporating the relevance vector machine technology. Their experimental results showed that this model has significant advantages in prediction accuracy, system stability, and execution efficiency [23].

Compared to traditional commercial software, these models show significant advantages in certain aspects. In terms of predictive accuracy, conventional water quality prediction software relies on fixed algorithms and predefined models, making it difficult to fully capture the complex nonlinear relationships and temporal dependencies in wastewater data. In contrast, machine learning models can autonomously learn and adapt to these complex patterns, providing more accurate and reliable prediction results for complex water quality data. Regarding data adaptability, the relatively rigid model structures of traditional commercial software limit their ability to adapt to different types of data. These software programs are usually only applicable to specific fields, such as DHI’s MIKE series for surface water systems [24] and EFDC for rivers, lakes, and reservoirs [25]. However, machine learning models can continuously optimise model parameters through training to adapt to the characteristics and variations of different water quality data, featuring a wide range of applications [26]. Commercial software also has its advantages. It usually comes with a more user-friendly interface, which is convenient for non-professional users to quickly get started, has a low usage threshold, and is suitable for routine water quality prediction tasks with relatively fixed patterns.

Deep learning models, as a subset of machine learning, leverages multi-layer neural networks to model complex data. Unlike traditional machine learning methods that often rely on manual feature extraction, deep learning automatically extracts features through multiple layers of processing [27]. This enables it to capture intricate patterns in large and complex datasets. These networks extract high-level features of the data layer by layer through multiple processing layers, thus enabling the model to learn complex patterns and relationships in the data [28,29,30]. In the field of wastewater treatment, deep networks are able to process many forms of data including time series and multidimensional sensor data for predicting water quality parameters, monitoring anomalies and optimising treatment processes. Owing to their robust learning capabilities and inherent flexibility, deep neural networks demonstrate exceptional proficiency in managing dynamically changing environments and addressing nonlinear challenges in wastewater treatment [31,32,33]. For example, Bekkari et al. proposed a deep learning technique to predict the COD of effluent from a WWTP for ten months using deep learning techniques, and the results showed that the neural network modelling approach achieved desirable prediction results and could provide an effective tool for the simulation, control, and prediction of WWTP operations [34]. Farhi et al. proposed a deep learning method based on LSTM for predicting the ammonia and nitrate concentrations in wastewater and achieved high accuracy and F1 scores [35]. Cheng et al. constructed a deep learning model based on LSTM and GRU to predict critical features in WWTPs. Moreover, in terms of efficiency, the GRU-based model converges faster than the LSTM-based model [36].

In addition, the performance of data-driven water quality prediction models is strongly dependent on the degree of compliance between the chosen modelling approach and the actual characteristics of the object under study [37,38]. Many existing studies have focused on building more realistic water quality prediction models and improving prediction accuracy by adapting model assumptions to the specific characteristics of the wastewater treatment process based on commonly used and well-established modelling approaches [39,40]. There is relatively little literature on the evaluation of the comprehensive effectiveness of these fundamental modelling approaches in practical applications, and most studies fail to explore the effectiveness of the models at the theoretical level. Furthermore, within the specific application contexts of IETPs, the existing models for predicting water quality in urban WWTPs demonstrate limited generalisability. Industrial effluent treatment, when compared to other process industries, possesses unique characteristics. Considering the numerous factors that may influence effluent quality, such as the quality and quantity of influent, process control variables, and energy consumption parameters, predicting the complex water quality in IETPs represents a formidable challenge [41].

This paper presents a data-driven approach based on multi-source data fusion for predicting effluent water quality in an IETP. The aim is to assess the existence of significant mapping relationships between the multi-source data of the IETP and the effluent water quality indicator data. And, we compare the performance of several common data-driven soft-measurement modelling approaches for online water quality prediction. Through this detailed comparative study, the effectiveness and limitations of these approaches in dealing with real wastewater data will be explored, providing important references and guidance for subsequent studies, especially in the selection of features and the optimisation of modelling techniques suitable for wastewater treatment prediction tasks.

## 2. Materials and Methods

### 2.1. The Research Framework

**Figure 1:**
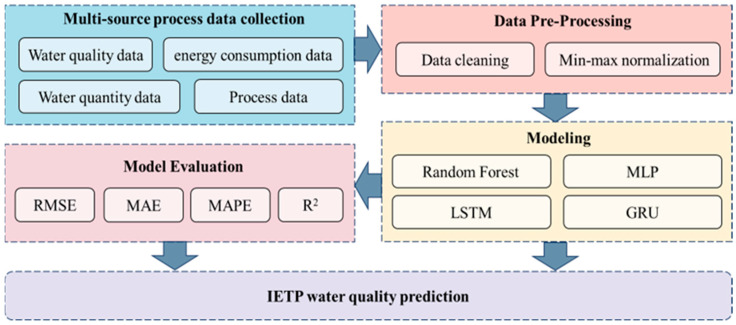
Framework of the proposed multi-source data fusion prediction model.

In this study, we introduce a research framework based on the fusion of multi-source data, designed for conducting comparative analyses of water quality in actual industry effluent treatment plant (IETP), as illustrated in Figure 1. The process is segmented into five distinct parts: (a) Initially, multi-source datasets within the IETP are collected; (b) These datasets are subsequently pre-processed to transform the data into a format suitable for computation; (c) Subsequently, the data are input into predictive models, which include machine learning models such as RF and deep learning models like MLP, LSTM, and GRU; (d) Finally, the performance of these models is evaluated to assess their efficacy.

### 2.2. Data Pre-Processing

The complex chemical processes in the IETP under study as well as the high failure rate of online data collection equipment and the limitations of storage technology lead to the possibility of missing data and outliers in the collected water quality data series, which may adversely affect the construction of the model. In this study, the K-nearest neighbours (KNN) method was used to fill the missing values in the original data [42]. The basic principle is to use the known values of the nearest K-neighbouring data points to estimate the unknown data point values based on the distance between the data points. The specific formula is as follows:

(1) $$X_{missing}=\frac{\sum_{i=1}^{K}w_{i}\cdot X_{i}}{\sum_{i=1}^{K}w_{i}}$$

where $X_{missing}$ represents the missing data values that need to be filled. K is the number of nearest neighbors selected, and $X_{i}$ is the data value of the i−th neighbor. The corresponding weight, $w_{i}$ is calculated through the formula $w_{i}=\frac{1}{d_{i}}$, where $d_{i}$ denotes the distance between this neighbour and the data point with the missing value.

Before feeding the data into the model, we applied a linear normalisation technique to ensure that all feature values are within the interval [0, 1]. This method helps to eliminate the scale difference between the features and reduce the problem of gradient explosion or disappearance that may occur during neural network training, thus improving the prediction performance of the model. The specific expression for normalisation is as follows:

(2) $${X^{*}}_{P}=\frac{X_{P}-X_{min}}{X_{max}-X_{min}}$$

where ${X^{*}}_{P}$ represents the normalised value of the P−th data point, falling within the interval [0, 1]. $X_{min}$ and $X_{max},$ respectively, denote the minimum and maximum values in the training set; $X_{P}$ is the original value of the P−th data point.

### 2.3. Predictive Model

#### 2.3.1. Random Forest (RF)

RF is an ensemble learning technique. Built upon multiple decision trees, the method improves generalisation and reduces the variance of the model by integrating the predictions from individual trees, and it has a certain level of robustness and accuracy [43]. Its nonlinear model structure enables the natural capture and modelling of nonlinear trends and seasonal patterns in the data. In the application of time series forecasting, RF can effectively handle high-dimensional data and capture complex relationships among variables. Therefore, RF is considered an effective tool in practical time series forecasting applications.

The construction of an RF involves the key following steps: Firstly, multiple sample sets are drawn randomly from the original dataset using the Bootstrap sampling technique, with each sample set used to train a decision tree. During the construction of the decision trees, the splitting of nodes is based on the optimal selection of features. This is typically accomplished by evaluating criteria such as information gain (IG) or Gini impurity.

(3) $$IG\left(D,A\right)=H\left(D\right)-\sum_{v\in Values\left(A\right)}\frac{\left|D_{v}\right|}{\left|D\right|}H\left(D_{v}\right)$$

where $H\left(D\right)\;$ represents the entropy of dataset D, and $D_{v}$ is the subset of data where the attribute A takes the value v.

(4) $$Gini\left(D\right)=1-\sum_{i=1}^{m}p_{i}^{2}$$

where $p_{i}$ represents the relative frequency of category i in dataset D.

**Figure 2:**
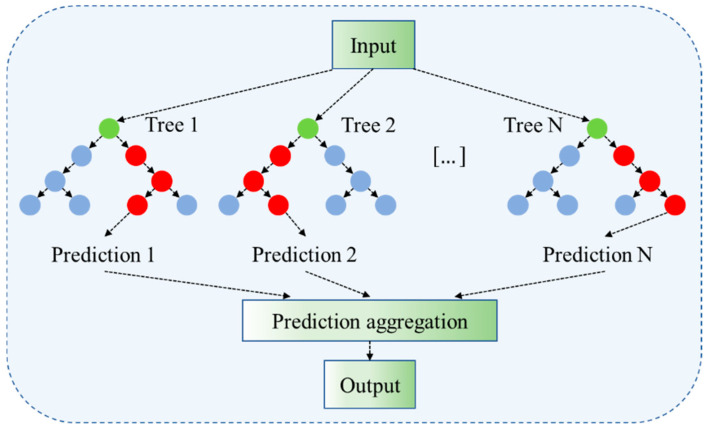
The diagram of the RF model.

Ultimately, the predictions of RF are obtained by averaging the outputs of all the decision trees:

(5) $${\hat{Y}}_{RF}\left(x\right)=\frac{1}{N_{trees}}\sum_{i=1}^{N_{trees}}f_{i}\left(x\right)$$

where ${\hat{Y}}_{RF}\left(x\right)$ represents the average output of all trees, which is the prediction result of the RF. $N_{trees}$ is the number of decision trees in the RF. $f_{i}\left(x\right)$ is the prediction result of the i−th decision tree. The RF model diagram is shown in Figure 2.

#### 2.3.2. Multilayer Perceptron (MLP)

**Figure 3:**
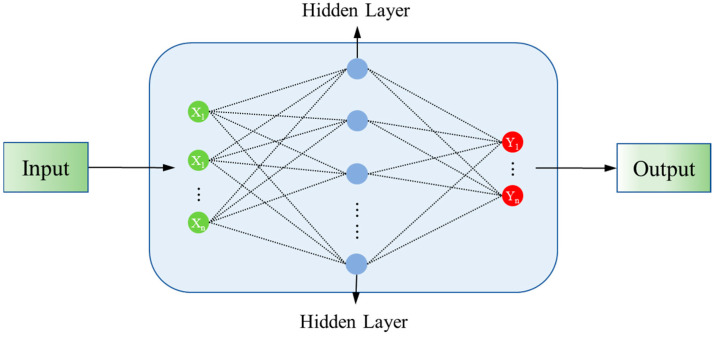
The diagram of MLP model.

The MLP is a neural network composed of multiple perceptron units, consisting of multiple layers of perceptron units, as shown in Figure 3. The MLP consists of an input layer, several hidden layers, and an output layer. Each layer consists of multiple neurons that interact with each other through weighted connections [44]. The core design feature of the MLP is the ability of its hidden layers to process the output through a nonlinear activation function, allowing the network to learn and approximate nonlinear and complex function mappings. This network structure has shown remarkable adaptability and efficacy in handling classification and regression problems. In MLP, the weights and biases are used as the trainable parameters of the network, which are optimised by stochastic gradient descent (SGD) method during the training process. The output of the neuron is calculated by the following equation:

(6) $$y_{i}=f\left(\sum_{j=1}^{n}\left(x_{j}\cdot w_{ij}+b_{i}\right)\right)$$

where $x_{j}$ represents the output of the j−th neuron, and n is the number of neurons in the current layer. $w_{ij}$ denotes the connection weights between neurons of adjacent layers, $b_{i}$ is the bias value of the neuron, and $y_{i}$ is the output of the i-th neuron. Neurons in the current layer serve as outputs for each connected neuron from the previous layer. The Sigmoid function, commonly employed as an activation function, is defined as:

(7) $$sigmoid\left(x\right)=\frac{1}{1+e^{-z}}$$

Its output is in the range of 0 to 1. It is mainly used in scenes where the output needs to be limited to a specific range.

#### 2.3.3. Long Short-Term Memory (LSTM)

**Figure 4:**
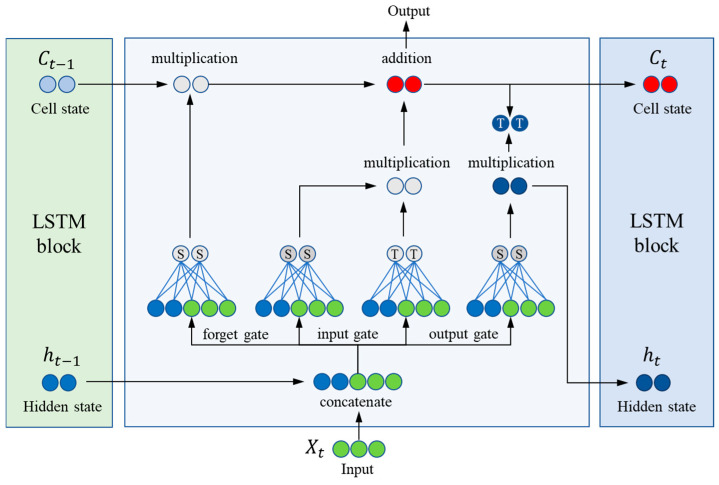
The diagram of LSTM model.

LSTM is a variant of recurrent neural networks (RNNs) specifically designed to address the problem of gradient vanishing or gradient explosion that standard RNNs may encounter when dealing with long-term data dependencies [45]. The key innovation of LSTM lies in its complex internal structure, shown in Figure 4, which consists of three distinctive gate control mechanisms: Forget Gate, Input Gate, and Output Gate as well as a unit to maintain long-term state. These mechanisms work in concert to precisely control the flow of information, enabling the network to efficiently capture and maintain long-term dependencies in sequential data. Each LSTM unit can be viewed as a miniature neural network module containing multiple control systems with gate control mechanisms that utilise nonlinear activation functions and element-by-element multiplication to determine whether information is retained or forgotten. The following is an operational and mathematical representation of the main components of the LSTM unit:

(1) Forget gate:

(8) $$f_{t}=\sigma \left(W_{f}\cdot \left[h_{t-1},x_{t}\right]+b_{f}\right)$$

where $f_{t}$ controls the amount of information transferred from the cell state at the previous moment $C_{t-1}$ to the current moment. The Sigmoid function *σ* ensures that the output values lie between 0 and 1, where $W_{f}$ and $b_{f}$ are the weight and bias of the forget gate, respectively.

(2) Input gate:

(9) $$i_{t}=\sigma \left(W_{i}\cdot \left[h_{t-1},x_{t}\right]+b_{i}\right)$$

(10) $${\tilde{C}}_{t}=tanh\left(W_{C}\cdot \left[h_{t-1},x_{t}\right]+b_{C}\right)$$

The input gate determines the extent to which the new input information ${\tilde{C}}_{t}$ influences the current cell state. Here, the activation function tanh is used to normalise the data to a range between −1 and 1.

(3) Unit status update:

(11) $$C_{t}=f_{t}*\times C_{t-1}+i_{t}\times {\tilde{C}}_{t}$$

The update of the cell state $C_{t}$ is achieved by forgetting the previous state and incorporating information from the new candidate state.

(4) Output gate:

(12) $$O_{t}=\sigma \left(W_{O}\cdot \left[h_{t-1},x_{t}\right]+b_{O}\right)$$

(13) $$h_{t}=O_{t}\times tanh\left(C_{t}\right)$$

The output gate controls the output portion of the current cell state $C_{t}$ to the next time step.

The training of LSTM involves forward propagation and gradient computation based on backpropagation, as well as parameter updating using optimisation algorithms similarly to other types of neural networks (e.g., SGD, Adam, etc.). Through this mechanism, LSTM is able to effectively learn the long-term dependencies present in time series data.

#### 2.3.4. Gated Recurrent Unit (GRU)

**Figure 5:**
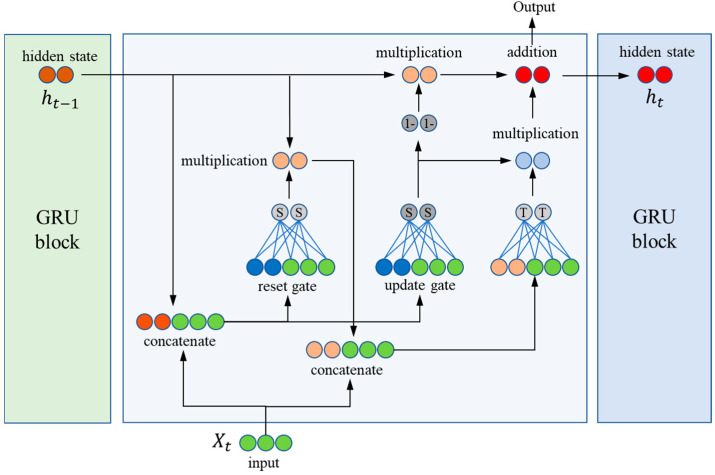
The diagram of GRU model.

GRU is an efficient neural network structure for long-term dependency problems in recurrent neural networks (RNNs). Similarly to LSTM, GRU controls the information flow through a special gating mechanism, but its structure is relatively more simplified [46]. The structure of GRU is shown in Figure 5, which usually only contains two gates: the update gate and reset gate. This simplified design not only reduces the number of parameters, but also improves the computational efficiency, which enables GRU to match or outperform LSTM in some tasks. The essence of GRU lies in how it integrates new input information with past memories through the gating mechanism, and the following are the main components of the GRU unit and their mathematical expressions:

(1) Update Gate:

(14) $$z_{t}=\sigma \left(W_{z}\cdot \left[h_{t-1},x_{t}\right]+b_{z}\right)$$

The update gate $z_{t}$ determines how much of the previous memory $h_{t-1}$ is retained. The σ sigma representation denotes the sigmoid function, ensuring that the output value is between 0 and 1, where $W_{z}$ and $b_{z}$ are the weight and bias of the update gate, respectively.

(2) Reset Gate:

(15) $$r_{t}=\sigma \left(W_{r}\cdot \left[h_{t-1},x_{t}\right]+b_{r}\right)$$

The reset gate $r_{t}$ determines how much of the previous memory is taken into account in the computation of the new candidate state. This can be thought of as determining how much past information is reset before the new output is computed.

(3) State update:

(16) $${\tilde{h}}_{t}=tanh\left(W_{h}\cdot \left[r_{t}{\cdot h}_{t-1},x_{t}\right]+b_{h}\right)$$

where, ${\tilde{h}}_{t}$ is the candidate new state, which is computed based on the reset past state $r_{t}{\cdot h}_{t-1}$ and the current input $x_{t}$. The tanh function helps to normalise the data between −1 and 1.

(17) $$h_{t}=\left(1-z_{t}\right)\cdot h_{t-1}+z_{t}\cdot {\tilde{h}}_{t}$$

The final hidden state $h_{t}$ is updated by combining the previous state $h_{t-1}$ with the newly computed candidate state ${\tilde{h}}_{t}$ and the weighing the effects of both by updating the gate $z_{t}$.

### 2.4. Strategies for Multi-Source Feature Fusion (RReliefF)

The RReliefF algorithm is an algorithm for feature structure optimisation on multi-source data [47], the core idea of which is to assess the importance of each feature pair by estimating the ability of that feature to distinguish between pairs of similar samples [48]. Based on this idea, the algorithm ranks the variables with higher weights implying higher importance. Specifically, there are implementation steps as follows: (1) A sample *x* is randomly selected from the dataset; (2) Find the *k* nearest similar samples (i.e., nearest similar neighbours) and *k* dissimilar samples (i.e., nearest dissimilar neighbours) to *x*; (3) For each feature *f*, update its weights. The update of the weights depends on the difference between the value of *x* on that feature and the value of its nearest neighbour on that feature. (4) Repeat the above steps several times, each time selecting a different sample *x*, and then calculate the average weight for each feature.

For each feature *f*, its weight update can be expressed as:

(18) $$\;\;\;W\left[f\right]=W\left[f\right]-\sum_{i=1}^{k}diff\left(f,x,{nearhit}_{i}\right)+\sum_{i=1}^{k}diff\left(f,x,{nearmiss}_{i}\right)$$

where $W\left[f\right]$ is the weight of feature *f*, $diff\left(f,x,{nearhit}_{i}\right)$ represents the differences between the feature *f* in instance *x* and the feature *f* in the *k* nearest hits (nearest neighbours of the same class). The function diff typically measures these difference. Contrarily, $diff\left(f,x,{nearmiss}_{i}\right)$ quantifies the differences between the value of feature *f* in instance *x* and the value of feature *f* in the *k* nearest misses (nearest neighbours of a different class).

### 2.5. Evaluation of Model Performance

In this study, four evaluation metrics were set to assess the experimental model, root mean square error (RMSE RMSE), mean absolute error (MAE), mean absolute percentage error (MAPE), and the coefficient of determination ($R^{2}$) to evaluate the prediction model to compare with the comparative experimental model.

(19) $$RMSE=\sqrt{\frac{1}{n}\sum_{t=1}^{n}{\left(y_{t}-\hat{y_{t}}\right)}^{2}}$$

(20) $$MAE=\frac{1}{n}\sum_{t=1}^{n}\left|\hat{y_{t}}-y_{t}\right|$$

(21) $$MAPE=\frac{100}{N}\left(\sum_{t=2}^{n}\frac{\hat{y_{t}}-y_{t-1}}{y_{t}}\right)$$

(22) $$R^{2}=1-\frac{\sum_{t=1}^{n}{\left(\hat{y_{t}}-\bar{y}\right)}^{2}}{\sum_{t=1}^{n}{\left(y_{t}-\bar{y}\right)}^{2}}$$

where n is the length of the time series, $\hat{y_{t}}$ denotes the predicted value, $y_{t}$ denotes the true value, and $\bar{y}$ is the average of the true values. MAE quantifies the average magnitude of errors in predictions, providing a direct measure of average error that is notably resilient to outliers. MAPE represents the error as a percentage of the actual values, offering an intuitive scale that reflects the error relative to the magnitude of the data being predicted. RMSE calculates the square root of the average of squared differences between predicted and actual values, significantly penalising larger errors and thereby underscoring the model’s performance on substantial discrepancies. $R^{2}$ gauges the proportion of variance in the dependent variable that can be predicted from the independent variables, thereby indicating the model’s explanatory power and overall effectiveness [49].

### 2.6. IETP

**Figure 6:**
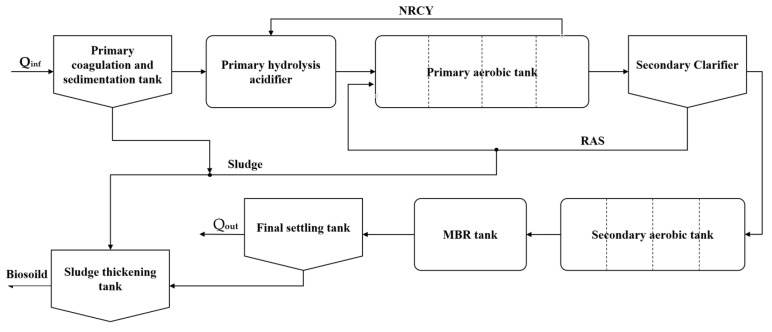
Flowchart of IETP treatment processes.

The data used in this study were obtained from an IETP located in the circular economy park of Huizhou District, Anhui Province, China. This IETP is designed to treat 5000 m^3^/d of industrial and domestic wastewater from the park. The treatment process consists of several stages, as shown in Figure 6, including primary coagulation and sedimentation, biological treatment, MBR technology, and final sedimentation, effectively treating wastewater through chemical and biological methods to ultimately produce clean effluent. In the primary sedimentation tank, coagulants like PAC and flocculants like PAM are dosed at concentrations adjusted to the incoming suspended solids to enhance their settling. After primary treatment, the secondary aerobic tank further degrades the residual dissolved organics, improving wastewater biodegradability and ensuring discharge compliance. The biological treatment system uses a combined process of “physicochemical precipitation + primary hydrolysis acidification + primary aeration + primary sedimentation + secondary aeration + MBR + final sedimentation − sludge thickening”, integrating multiple treatment units to efficiently remove organics, NH_3_-N (with over 90% removal) and TN (around 80% removal). The IETP processes daily up to 5000 m^3^ of wastewater from various chemical plants, with a retention time of 30.5 h. The treated effluent stably meets the tertiary standards of the “Integrated Wastewater Discharge Standard” (GB8978-1996). The IETP generates about 2 m^3^ of biosolids daily, which are handled by a third-party company with professional qualifications.

### 2.7. Statistical Information on Data

This study collected data from the online monitoring system of an IETP, spanning from 1 January 2022 to 31 December 2022. A total of 8689 sets of data were gathered. Data were collected hourly and primarily included four main categories encompassing 17 sets in total: flow data, water quality data, process data, and energy consumption data, which can be described as $X={\left(x^{1},x^{2},{\ldots ,x}^{n}\right)}^{T}=\left(x_{1},x_{2},\ldots ,x_{t}\right)\in R^{n\times t}$. The main statistical features (mean, median, standard deviation, and coefficient of variation) and interpretations of the data are provided in Table 1. The analysis of effluent COD (COD_eff_), effluent NH_3_-N (NH_3_-N_eff_), effluent TN (TN_eff_), and effluent TP (TP_eff_) demonstrates significant fluctuations and non-linear characteristics. These features differ markedly from those of urban WWTPs, posing greater challenges in establishing accurate predictive models.

### 2.8. Model Development

For the training process of all the models, the multi-source data from the IETP were divided into training, validation, and testing sets in the ratio of 7:2:1. In the training phase, the models are mainly learnt from the training set to fit the features and patterns of the data, and the testing phase is used to evaluate the performance of the models on completely unseen data and to assess their generalisation ability. Among the four different types of models RF, MLP, LSTM and GRU. Water quantity, process variables (pH, ORP, and DO), energy consumption data (Blower flow rate and Blower current), and water quality data (COD, NH_3_-N, TN, and TP) 17 were used as inputs, and effluent water quality data (COD_eff_, NH_3_-N_eff_, TN_eff_, and TP_eff_) were used as the outputs, respectively.

In order to maximise the accuracy of each prediction model, this study adopts the parameter grid search method to optimise the tuning parameter [50], and the specific optimal parameter set is shown in Table 2. In order to ensure the consistency of the performance of the deep learning models, we uniformly set the learning rate to 0.001. In addition, all of the prediction models in this study were run on a NVIDIA RTX 4060ti GPU (NVIDIA, Santa Clara, CA, USA) equipped with 2.6 GHz CPU and a computing system and were developed and implemented using the Python3.8 language.

## 3. Results and Discussion

### 3.1. Evaluation of Prediction Results and Model Performance

**Figure 7:**
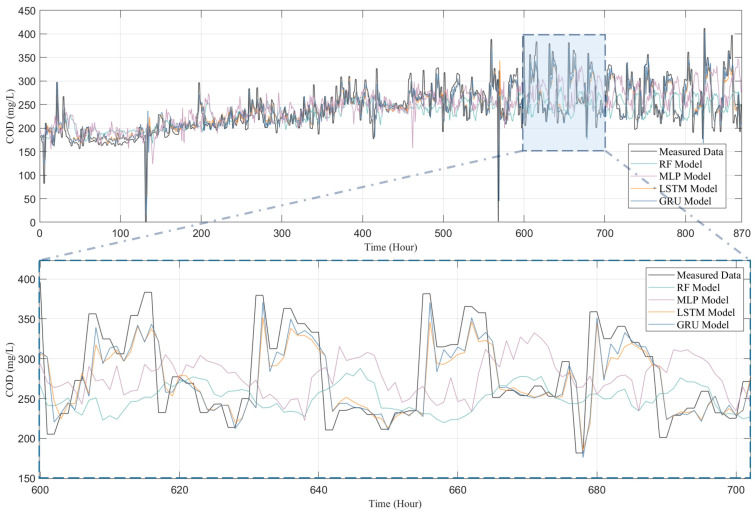
Comparison of COD predicted by RF model, MLP model, LSTM model and GRU model with measured data.

**Figure 8:**
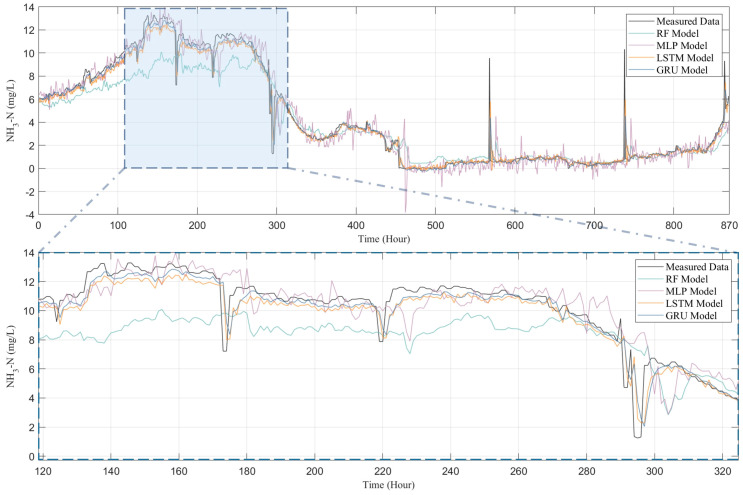
Comparison of NH_3_-N predicted by RF model, MLP model, LSTM model, and GRU model with measured data.

**Figure 9:**
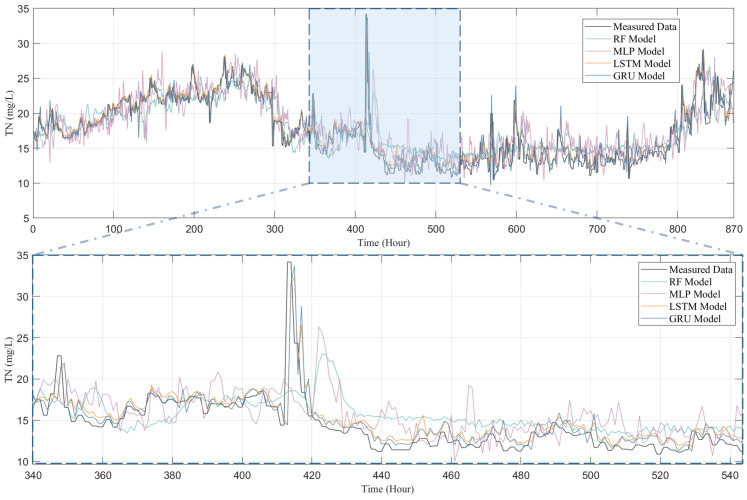
Comparison of TN predicted by RF model, MLP model, LSTM model, and GRU model with measured data.

**Figure 10:**
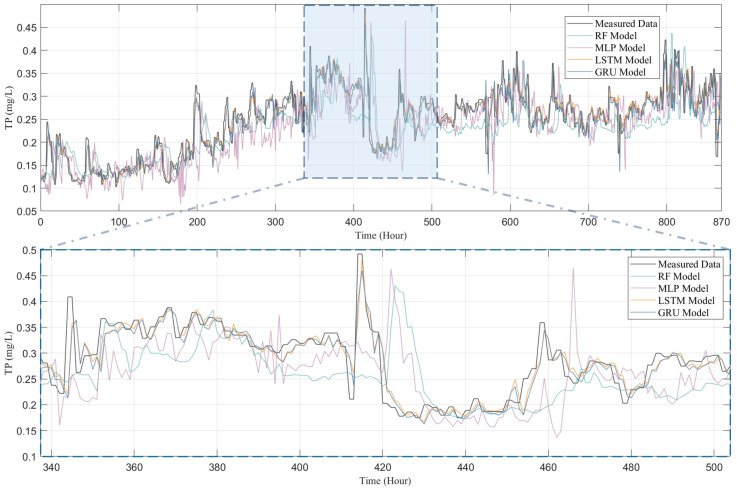
Comparison of TP predicted by RF model, MLP model, LSTM model, and GRU model with measured data.

To thoroughly examine the performance of four models—RF, MLP, LSTM, and GRU—in predicting water quality, this study conducted a comparative analysis between the predicted outcomes for four water quality indicators (i.e., COD, NH_3_-N, TN, and TP) and their actual observational data, with the results presented in Figure 7, Figure 8, Figure 9 and Figure 10. Figure 7 presents both the actual observation data and the predicted outcomes for effluent COD. It is evident that the effluent COD concentration fluctuates around 200 mg/L, which can be attributed to the factory’s requirement to comply with the third-level standard set forth in the “Integrated Wastewater Discharge Standard” (GB8978-1996), stipulating that COD levels must not exceed 300 mg/L. Furthermore, a noticeable fluctuation in the effluent COD concentration occurred after 500 h of operation, which can be attributed to significant variations in the influent COD parameters. These fluctuations in the influent COD contributed to the overall instability of the effluent COD concentration in the wastewater treatment plant. Nevertheless, all models successfully captured the general trend of the COD fluctuations.

Although all models, including RF, MLP, LSTM, and GRU, managed to capture the general trends of the data to some extent, the RF and MLP models show significant shortcomings in terms of predictive accuracy and response timeliness. Particularly, the RF model performed poorly in predicting COD and TP, as shown in Figure 7 and Figure 10, failing to effectively track the data’s variability. The MLP model, while showing slight improvements in certain areas, significantly overestimated peak concentrations in TN predictions (as depicted in Figure 9), indicating a tendency towards overfitting; it tends to underestimate the peak values in COD predictions, further highlighting its disadvantages in handling complex time-series data in IETP.

**Figure 11:**
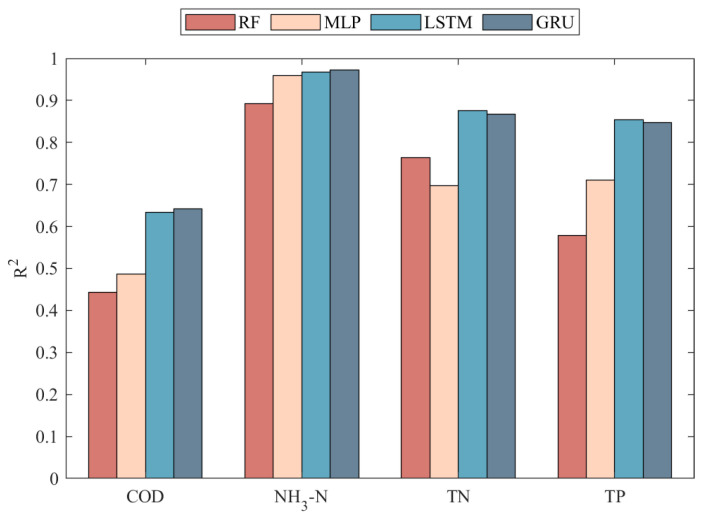
Visualisation of accuracy assessment for RF, MLP, LSTM, and GRU models.

In contrast, LSTM and GRU demonstrate exceptional predictive performance, with a high R^2^ value shown in Figure 11. In the highly variable data characteristics of COD, TN, and TP (as shown in Figure 7, Figure 8 and Figure 10), both models not only effectively track peaks and troughs but also exhibit high accuracy in capturing critical turning points. When handling the relatively more stable but complex NH_3_-N data, as in Figure 9, LSTM and GRU precisely fit the data curves, showing their sensitivity to subtle data variations and rapid response to trend changes. This excellent predictive outcome is attributed to the inherent advantages of these models in handling time-series data, such as their unique gating mechanisms, which provide efficiency in processing long-term dependency information. The GRU, with fewer parameters and faster convergence than the LSTM, is particularly apt for predicting water quality in wastewater treatment systems. This simplification of parameters not only enhances the computational efficiency of the model but also helps reduce the risk of overfitting, thus achieving faster and more stable predictive performance in practical applications.

Figure 11 illustrates the predictive performance of different models across various water quality indicators. LSTM and GRU models demonstrate superior predictive capabilities compared to RF and MLP. Nevertheless, for COD, all models underperform relative to TP, TN, and NH_3_-N. Even advanced LSTM and GRU models only achieve an R^2^ of approximately 0.65, while RF and MLP yield R^2^ values between 0.4 and 0.5. This is due to the fact that COD, a comprehensive indicator of organic water pollution, is influenced by a variety of factors, including the source, composition, flow rate, and treatment processes of wastewater. These factors interact in complex ways, leading to the high complexity and nonlinearity of COD. In contrast, TP, TN, and NH_3_-N display relatively simple and linear variation patterns, making them easier for models to capture and resulting in higher predictive accuracy.

### 3.2. Comparison of the Models

To evaluate the predictive accuracy of the four models across the four effluent quality indicators (i.e., COD, NH_3_-N, TP and TN), a comprehensive analysis was conducted using the RMSE, MAE, MAPE, and R^2^ assessment metrics, as detailed in Table 3. Taking the performance of the RF model as a benchmark, in the prediction of COD, the R^2^ of the MLP model improves by 9.68%, 7.51%, and 22.83% in the prediction of COD, NH_3_-N, and TP, respectively, which reflects the fact that the deep learning technique demonstrates a better ability to fit data and explain the variability when dealing with highly nonlinear systems compared with the traditional machine learning methods. However, the R^2^ of MLP decreases by 8.77% in the prediction of TN, which may stem from the specific characteristics of the TN, such as higher noise levels or particular data distributions, which are incompatible with the learning mechanism of MLP, leading to the overfitting of the model on this metric (as depicted in Figure 9).

Compared with the MLP model, the LSTM model shows significant advantages in terms of prediction accuracy. Specifically, the LSTM model improves the R^2^ of the prediction results by 30.18%, 0.83%, 25.68%, and 20.28% for COD, NH_3_-N, TN, and TP, respectively, with a significant decrease in RMSE. This performance advantage is mainly attributed to the high performance of LSTM in processing time-series data, especially its gating mechanism which provides strong support for the fine-grained management of information, thus effectively capturing the time-dependence and long-term dependencies in the data. In addition, compared to LSTM, the GRU model slightly outperforms in COD and NH_3_-N prediction. This is demonstrated by the fact that the RMSE of the GRU model is reduced by 1.02% and 7.03% for these two parameters, while the MAE is reduced by 5.91% and 20.32%, respectively. This further demonstrates that the GRU is able to reach convergence faster when dealing with specific types of IETP time series data due to its more compact structure, thus providing higher forecasting efficiency and accuracy in some cases.

Overall, deep learning algorithms usually outperform traditional machine learning techniques in terms of performance. Among the deep learning frameworks, the GRU model demonstrated superior performance in most cases, especially in the prediction of COD and NH_3_-N parameters. In contrast, LSTM is slightly better in terms of TN and TP prediction accuracy, although the difference in performance is not significant.

### 3.3. Further Understanding Changes in Effluent Quality Through RreliefF

**Figure 12:**
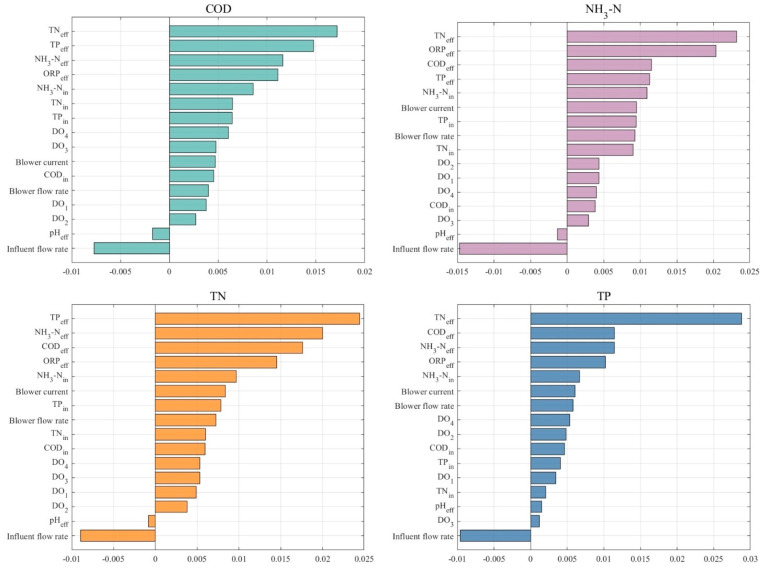
Feature importance ranking based on the RReliefF algorithm.

In order to explore in depth the relationship between the input model variables and the target output variables, the RReliefF analysis methodology described in Section 2.4 is employed in this study to evaluate the GRU prediction model used. The RReliefF values quantify the extent to which each input variable contributes to the target output, where higher values indicate that the variable contributes more significantly to the model output. Figure 12 illustrates the RReliefF values for each input variable, ranked in descending order, revealing the extent to which the four effluent indicators are influenced by multiple sources of data, including water quantity data, process variables, energy consumption data, and water quality data. Based on the RReliefF values, the study sets three further threshold intervals: −0.02 to 0, −0.02 to 0.005, and 0.005 to 0.03. These three intervals represent the removal of the variables that negatively affect the predicted targets, those that have less impact on the predicted targets, and those that have the greatest impact on the predicted variables, respectively.

In the prediction task for the four water quality indicators, removing the variables that have the greatest impact on the predicted variables (RReliefF values of 0.005 to 0.03) increases the RMSE values of the experimental data by 31.62% to 104.19%, increases the MAE by 42.30% to 138.60%, and decreases the R^2^ values by 53.74% to 87.66%. This means that the removal of more sensitive variables significantly reduces the performance of the effluent water quality prediction model. Based on the analysis in Figure. 4, it can be observed that there is a significant interaction between the effluent water quality indicators on the prediction results. This phenomenon may reflect the treatment efficiency of the IETP. This suggests that the interactions between the water quality indicators are crucial for understanding and optimising the treatment process. In addition, the predicted results for NH_3_-N_eff_, TN_eff_ and TP_eff_ show high sensitivity to ORP and energy consumption data. This suggests that these water quality parameters are closely related to the redox conditions and energy consumption of the treatment process. This correlation may indicate that monitoring these indicators may be particularly important when adjusting the treatment process to optimise efficiency and reduce energy consumption. In the experiment of removing variables with small effects on the prediction targets (RReliefF values of −0.02 to 0.005), it is found that R^2^ was significantly reduced by 14.20% in the prediction task of COD_eff_, indicating that even the removal of the variables with small effects negatively affected the prediction accuracy of COD_eff_, compared to that of the NH_3_-N_eff_, TN_eff,_ and TP_eff_ prediction tasks, the removal of these variables had almost no effect on the model’s prediction results, and the change in R^2^ is less than 0.6% for all of them, which implies that the IETP for the prediction of effluent COD needs richer parameter support. After removing the input variables that negatively affected the prediction objectives, it is observed that the R^2^ of the model improves by 0.41–1.03%, the RMSE decreases by 0.49–2.72%, while the MAE decreases by 0.67–10.67%. This suggests that removing these variables resulted in an improvement in model performance, which, although not significant, verifies that model performance is not negatively affected and may even improve slightly. From this result, it can be seen that removing variables that do not contribute or even negatively affect the model output based on the results of the RReliefF analysis can both improve the efficiency of the model and help optimise the monitoring and management strategy of the IETP, allowing resources to be further focused. See Table 4.

## 4. Conclusions

In this study, a deep learning framework based on multi-source data fusion was developed with the aim of accurately predicting the effluent quality of an IETP. Through a case study of an IETP in Anhui Province, China, this paper compares the efficacy of four models, namely RF, MLP, LSTM, and GRU, in predicting water quality metrics such as COD, NH_3_-N, TN, and TP. It is found that the deep learning models, especially LSTM and GRU, significantly outperform the traditional machine learning models (RF and MLP) in terms of prediction accuracy and generalisation ability. LSTM and GRU greatly improve the prediction accuracy by effectively capturing the long-term dependencies in the time-series data and dealing with the nonlinearity and high variability of the data. In particular, these models demonstrate excellent performance in the prediction of highly variable indicators such as COD, TN, and TP.

In addition, this study applied the RReliefF algorithm for feature importance analysis, successfully identifying key variables that influence effluent quality, such as Tneff, Tpeff, and CODeff. These findings offer substantial support for model optimisation and the operational management of IETPs. In terms of model optimisation, increasing the monitoring frequency and accuracy of these key variables can improve the quality of input data, thereby enhancing the predictive performance of the model. The identification of key variables is equally significant in the operational management of IETPs. These variables enable management personnel to monitor the wastewater treatment process in real-time. Based on this information, they can adjust process parameters and chemical dosages promptly, improving treatment efficiency, reducing costs, and ensuring stable and compliant effluent quality. Furthermore, the monitoring of these key variables aids in the development of an early warning system. This system allows for the timely detection of potential anomalies or equipment failures. It enables management personnel to take proactive measures, prevent the occurrence of major system accidents, and ensure the stability and effectiveness of the wastewater treatment process.

This study not only confirms the effectiveness of deep learning techniques in dealing with complex environmental problems, but also lays a theoretical and practical foundation for the future implementation of intelligent and automated technologies in the real water treatment industry. Future research will aim to extend and refine the findings of this study. Specifically, we intend to incorporate a wider variety of data sources, such as meteorological and process operation data, to more comprehensively capture the factors that influence the wastewater treatment processes. Additionally, we will undertake on-site testing at operational water treatment plants to facilitate the integration of the model with existing control systems, thereby enabling intelligent control and automated decision support.

## Figures and Tables

**Table 1:** The main statistical features and interpretations of the data.

Measurement	Mean	Median	STD	CV	Comments
Influent flow rate	153.33	128	112.009	0.88	Instantaneous influent flow
COD_in_	1169.24	1240.2	656.726	0.53	Influent chemical oxygen demand-—mg/L
NH_3_-N_in_	7.8	1.89	15.021	7.95	Influent ammonia nitrogen—mg/L
TP_in_	0.87	0.66	0.61	0.92	Influent total phosphorus—mg/L
TN_in_	23.99	19.22	17.278	0.90	Influent total nitrogen—mg/L
pH_eff_	7.8	7.84	0.321	0.04	Effluent potential of hydrogen—mg/L
ORP_eff_	22.53	22.8	3.768	0.17	Effluent oxidation-reduction potential—mg/L
DO_1_	3.27	3.27	0.307	0.09	Dissolved oxygen in the primary aerobic tank—mg/L
DO_2_	3.27	3.27	0.35	0.11	Dissolved oxygen in the primary aerobic tank—mg/L
DO_3_	1.18	0.57	1.382	2.42	Dissolved oxygen in the secondary aerobic tank—mg/L
DO_4_	1.43	0.6	1.753	2.92	Dissolved oxygen in the secondary aerobic tank—mg/L
Blower flow rate	109.1	109.7	16.096	0.15	Air blower flow rate—m^3^/h
Blower current	254.88	261.7	44.311	0.17	Blower current—A
COD_eff_	234.45	234.8	41.541	0.18	Effluent chemical oxygen demand—mg/L
NH_3_-N_eff_	4.75	2.86	4.89	1.71	Effluent ammonia nitrogen—mg/L
TP_eff_	0.25	0.2	0.172	0.86	Effluent total phosphorus—mg/L
TN_eff_	22.48	21.69	7.8	0.36	Effluent total nitrogen—mg/L

**Table 2:** The specific optimal parameter set of four models.

Model	Parameters	Value
RF	Min samples leaf	16
	Min samples split	2
	Max depth	5
	Estimators	100
MLP	Number of layers	3
	Number of neurons	50, 10
	Activation	Identity
	Batch size	512
	Optimiser	adam
LSTM	Number of layers	2
	Number of neurons	128, 128
	Activation	Relu
	Batch size	512
	Optimiser	adam
GRU	Number of layers	2
	Number of neurons	128, 128
	Activation	Relu
	Batch size	512
	Optimiser	adam

**Table 3:** The predictive accuracy of the four models across the four effluent quality indicators.

Water Quality Indicators	Model	Evaluation Index			
		RMSE	MAE	MAPE	R^2^
COD	RF	41.349	28.913	62.450%	0.444
	MLP	39.715	28.688	62.681%	0.487
	LSTM	33.385	19.825	52.255%	0.634
	GRU	33.045	18.653	49.551%	0.642
NH_3_-N	RF	1.398	0.881	/	0.892
	MLP	0.861	0.508	/	0.959
	LSTM	0.768	0.364	/	0.967
	GRU	0.714	0.290	/	0.972
TN	RF	2.134	1.588	10.205%	0.764
	MLP	2.418	1.829	11.487%	0.697
	LSTM	1.562	0.994	6.301%	0.876
	GRU	1.614	0.938	5.811%	0.867
TP	RF	0.046	0.036	13.716%	0.578
	MLP	0.038	0.028	11.472%	0.710
	LSTM	0.027	0.016	6.832%	0.854
	GRU	0.027	0.017	6.805%	0.848

**Table 4:** Evaluating the prediction accuracy through decremental experiments.

Water Quality Indicators	Modelling Scenario	Feature Quantity	Evaluation Index		
			RMSE	MAE	R^2^
COD	A	16	33.045	18.653	0.642
	B	14	32.883	18.528	0.649
	C	8	35.234	20.193	0.550
	D	8	43.283	26.366	0.163
NH_3_-N	A	16	0.714	0.290	0.972
	B	14	0.695	0.259	0.981
	C	9	0.710	0.284	0.974
	D	7	1.418	0.618	0.454
TN	A	16	1.614	0.938	0.867
	B	14	1.597	0.927	0.874
	C	12	1.623	0.942	0.862
	D	4	3.051	2.101	0.108
TP	A	16	0.0270	0.0170	0.848
	B	15	0.0269	0.0168	0.851
	C	8	0.0269	0.0184	0.850
	D	8	0.0416	0.0301	0.392

Note: Scenario A: Predictive Modelling by GRU using all input variables; Scenario B-D: Predictive modelling by GRU with parameters excluded based on RReliefF values ranging from −0.02 to 0, −0.02 to 0.005, and 0.005 to 0.03, respectively.

## Data Availability

The data that support the findings of this study are available from the corresponding author upon reasonable request.
